# Dynamics of the transcriptome during chicken embryo development based on primordial germ cells

**DOI:** 10.1186/s13104-020-05286-w

**Published:** 2020-09-18

**Authors:** Aleksandra Dunislawska, Agata Szczerba, Maria Siwek, Marek Bednarczyk

**Affiliations:** Department of Animal Biotechnology and Genetics, UTP University of Science and Technology, Bydgoszcz, Poland

**Keywords:** Gene expression, Primordial germ cells, Microarray, White Leghorn

## Abstract

**Objective:**

Regulation of gene expression during embryo development on the basis of migration of primordial germ cells (PGCs) in vivo has been rarely studied due to limited cell number and the necessity to isolate PGCs from a large number of embryos. Moreover, little is known about the comprehensive dynamics of the transcriptome in chicken PGCs during early developmental stages. The current study investigated transcriptome dynamics of chicken PGCs at key developmental stages: 4.5, 8 and 12 days of embryo incubation. PGCs were collected, and RNA was isolated using a commercial kit for single cells. The isolated RNA was subjected to microarray analysis (Agilent Technologies).

**Results:**

Between 8 and 12 days of incubation, the highest number of genes was regulated. These data indicate that the most intense biological activity occurs between 8 and 12 days of embryo development. Heat map showed a significant decrease in gene expression on day 8, while it increased on day 12. The development of a precise method to isolate bird PGCs as well as the method to isolate RNA from single cells isolated from one embryo allows for early molecular analysis and detection of transcriptome changes during embryonic development.

## Introduction

Primordial germ cells (PGCs) are the earliest recognisable precursors of adult germ cells. They transfer the genetic information to the next generation of cells. Chicken PGCs originate from the epiblast [1]. They are located at the centre of the area pellucida at stage X of Hamburger-Hamilton (HH) [2] and are translocated anteriorly to the germinal crescent [3]. Subsequently, the PGCs localise in the vascular system and use those extraembryonic blood vessels as a vehicle to reach the germinal ridges (Additional file 1: Figure S1). They accumulate in germinal ridges as gonadal PGCs (gPGCs), are also termed as gonocytes [4] and differentiate into spermatogonia in males or oogonia in females. Circulating PGCs and gPGCs are functionally very similar. As precursors of reproductive cells, PGCs are an important tool in the study on reproduction of vertebrates and in gene expression and epigenetic studies. The first challenge in gaining knowledge and understanding of the mechanisms associated with PGCs is to analyse the changes that may occur in a given location in the embryo on the basis of PGCs from a given place, regardless of gender. PGCs migrate into the bloodstream before they reach the gonads and are classified as circulating PGCs (cPGCs). However, the regulation of gene expression during the migration and proliferation of PGCs in vivo has been rarely studied due to limited cell number. Little is known about the comprehensive dynamics of the transcriptome in chicken PGCs isolated from a single embryo during early developmental stages. At individual stages of development, the expression of only a limited number of genes was analysed [5, 6]. The present study aimed to analyse transcriptome dynamics of gPGCs at three developmental stages: day 4.5, 8 and 12 of embryo incubation.

## Main text

### Methods

#### Isolation of gPGCs

45 White Leghorn fertilized eggs were incubated at 37.8 °C for 4.5, 8 and 12 days HH stages: 26, 34 and 38, respectively [7], 15 eggs for time point to obtain embryos of suitable developmental stage. The gonads were cultured for up to 90 min at 37.8 °C in phosphate buffered saline without Ca^2+^ and Mg^2+^ (PBS [−]) according to Nakajima et al. [8] to obtain live gPGCs. PGCs were collected in lysis buffer, and RNA was isolated using a commercial kit (GenElute Single Cell RNA Purification Kit, Sigma Aldrich, Missouri, USA). To confirm that the RNA was derived from PGCs, PGC-related markers (*NANOG* and *DAZL*) were assessed by reverse transcription- quantitative polymerase chain reaction (RT-qPCR) based on the protocol described by Dunislawska et al. [9]. Primer sequences were derived from literature data [10].

#### Whole transcriptome analysis

Microarray analysis was performed using SurePrint G3 Custom GE 8 × 60 k microarrays. Three slides with four matrices each were used. The analysis for one time point was performed in four independent biological replicates. The microarray procedure was performed according to the manufacturer’s protocol for One-Color Microarray-Based Gene Expression Analysis (Agilent Technologies, Santa Clara, USA). The obtained data were analysed by GeneSpring GX software (Agilent Technologies). The following criteria were used to generate the gene lists: statistical significance (*P* value) higher than 0.05 and cut-off greater than 2.0 as upregulated genes and smaller than − 2.0 as downregulated genes. Statistical analysis was performed using one-way ANOVA. Selected time points were compared with each other. For qualitative analysis, Venn diagrams (bioinformatics.psb.ugent.be/webtools/Venn/) and a heat map were used to present the general direction of changes in the expression level. GeneSpring software was also used for qualitative assessment to assign genes to the main terms of gene ontology (GO). To analyse the interaction between individual proteins encoded by genes that showed changes in the expression level, an extended functional analysis was performed using the STRING software [11].

#### Microarray validation

A panel of eight high differentially expressed genes (DEGs) was selected for microarray validation by using RT-qPCR as described in Dunislawska et al. [12]. Two reference genes were used: *ACTB* [13] and *G6PDH* [14]. Sequences of primers for validation were designed based on cDNA nucleotide sequence (Additional file 1: Table S1) using NCBI Primer-Blast [15]. The relative gene expression analysis was performed using the ddCt method [16]. The significance of the gene expression data was determined with Student’s *t* test (*P* < 0.05).

### Results

#### Isolation of gPGCs

RT-qPCR confirmed that the isolated RNA was derived from PGCs. Results from the amplification curve and threshold cycle (Ct) values do not differ significantly between the developmental stages. The average Ct value for the *NANOG* gene is 27.4 while Ct of *DAZL* is 26. The results of *NANOG* and *DAZL* amplification are shown in the Additional file 1: Figure S2 and S3.

#### Quantitative analysis of DEGs

The total number of DEGs between 4.5 and 8 days of embryo development was 403 upregulated genes and 988 downregulated genes. There were 5304 upregulated genes and 2535 downregulated genes between 8 and 12 days of embryo development. The number of DEGs between 4.5 and 12 days of embryo development was 3571 upregulated genes and 1528 downregulated genes. The results are presented in Venn diagrams in Additional file 1: Figures S4 and S5.

**Fig. 1:**
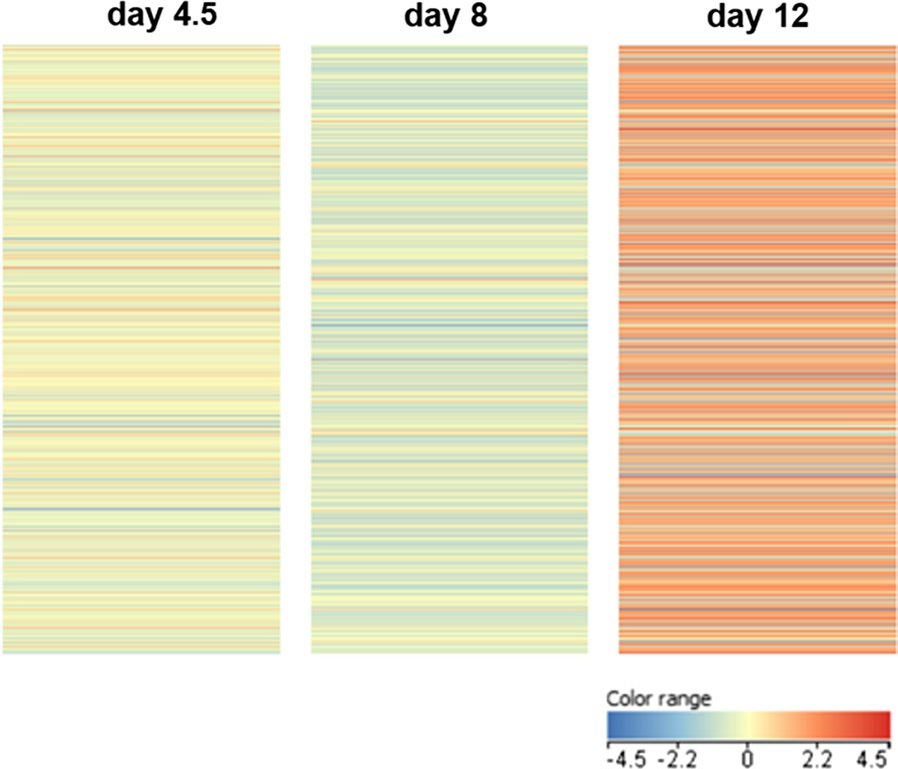
Individual values (heat maps) of genes expressed on day 4.5, 8 and 12

Heat maps (Fig. 1) show that on day 4.5 of embryo development, the expression level of the vast majority of genes was in the range of − 2.2 to 2.2. On day 8 of embryo development, the expression level of the majority of genes decreased. In contrast, gene expression was significantly upregulated on day 12 of embryo development.

#### Qualitative analysis of DEGs

**Fig. 2:**
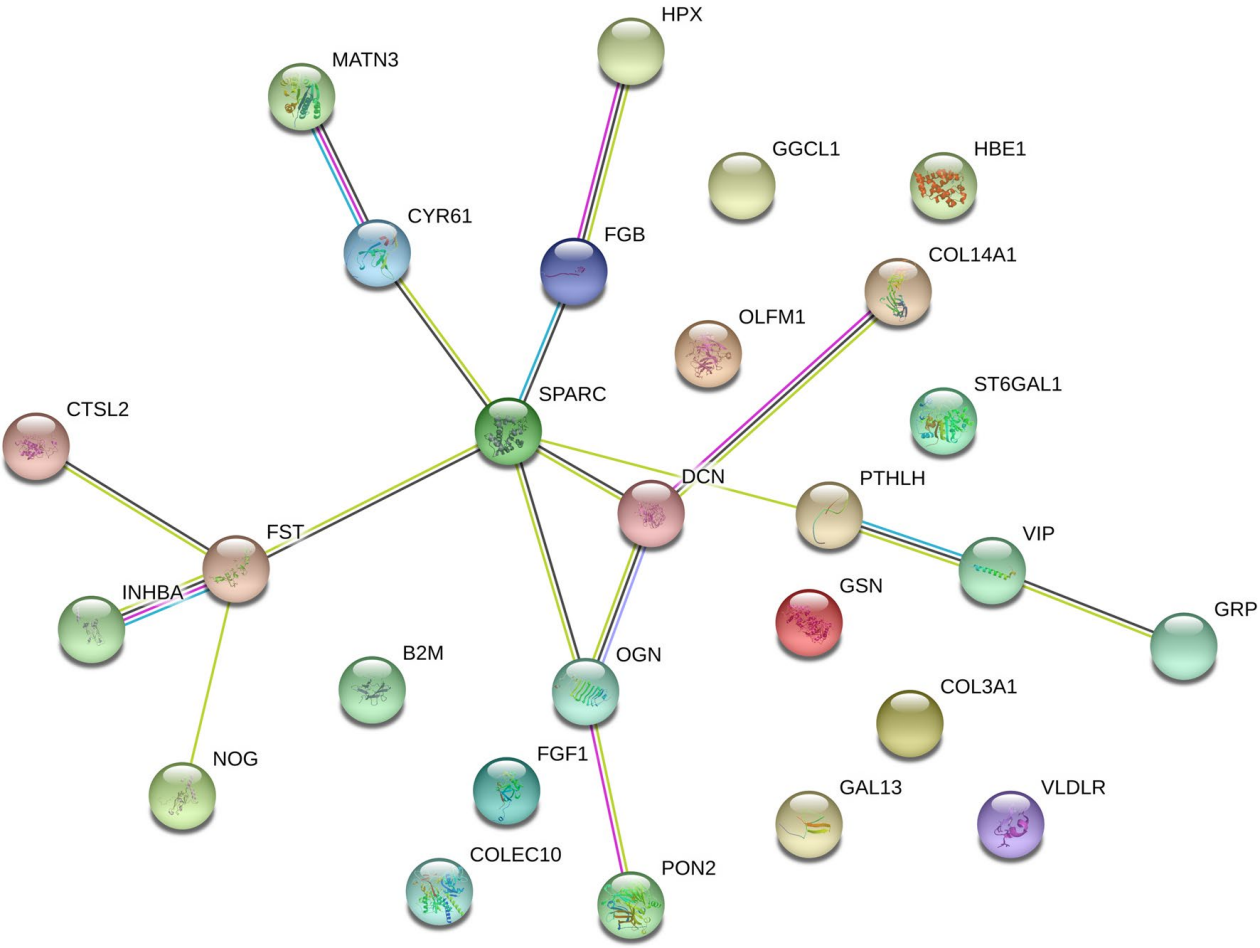
Analysis of the relationship between proteins encoded by genes whose expression was upregulated in PGCs on day 8 of embryo development. Lines of interactions according to STING software: light blue–from curated databases; pink–experimentally determined; dark green–gene neighborhood; red–gene fusions; dark blue–gene co-occurrence; light green–textmining; black–co-expression; violett–protein homology

The results are presented only for statistically significant (*P *< 0.05) GO terms and show a comparison of gene expression on day 8 and 12 versus that on day 4.5. The analysis of GO terms showed that the genes upregulated on day 8 were allocated to the GO term related to the extracellular region (GO:0005576). The relationship between these genes is shown in Fig. 2.

The downregulated genes on day 8 were assigned to the following GO terms: cytokine receptor binding (GO:0005126) and defence response to bacterium (GO:0042742 and GO:0042830). The relationships between these genes are shown in Additional file 1: Figure S6. The highest number of genes was upregulated on day 12 (detailed data are presented in Table 1). The genes downregulated on day 12 are mostly involved in organic cyclic compound binding (GO:0097159) and heterocyclic compound binding (GO:1901363).

**Table 1:** Gene Ontology terms based on the upregulated genes on day 12 in PGCs

GO ACCESSION	GO term	Total count
GO:0032501	Multicellular organismal process	719
GO:0044707		
GO:0050874		
GO:0032502	Developmental process	662
GO:0044767		
GO:0048856	Anatomical structure development	634
GO:0007275	Multicellular organism development	587
GO:0071944	Cell periphery	462
GO:0005886| GO:0005904	Plasma membrane	447
GO:0023052	Signaling	446
GO:0023046		
GO:0044700		
GO:0007165	Signal transduction	423
GO:0023033		
GO:0005576	Extracellular region	308
GO:0044459	Plasma membrane part	258
GO:0044421	Extracellular region part	232
GO:0005615	Extracellular space	212
GO:0003008	System process	155
GO:0031226	Intrinsic component of plasma membrane	146
GO:0005887	Integral component of plasma membrane	140
GO:0006811	Ion transport	132
GO:0050877	Nervous system process	96
GO:0007600	Sensory perception	57
GO:0007601	Visual perception	33
GO:0050953	Sensory perception of light stimulus	33
GO:0006936	Muscle contraction	22

#### Microarray validation

A panel of genes was selected on the basis of the highest and lowest values of fold change on microarray. A comparison of RT-qPCR validation and microarray results is presented in the Additional file 1: Table S2. The expression patterns of the majority of these genes (6 out of 8) were consistent between RT-qPCR and microarray.

## Discussion

The present study aimed to analyse the transcriptome of chicken PCGs at three different time points of embryo development. The motivation for this investigation was to show how quantitative gene expression in PGCs changes during chicken embryonic development. An extensive knowledge on avian PGCs has been generated from in vitro studies [17–19]. In contrast, there is limited information on the regulation of gene expression during the migration of PGCs in vivo. This is mainly because of the limited number of available PGCs [20]. The new technique for isolating viable avian PGCs combined with the method of RNA isolation from a single cell offers a unique approach for the study of cell transcriptome dynamics. In the current study, both techniques were combined together for the first time. We isolated PGCs in vivo directly from the embryo and subjected them to the transcriptome analysis of RNA isolated from single cells. Earlier studies were based on in vitro cultures [21] and thus do not reflect the complexity of living organisms. The objective of these studies was to obtain PGCs from a large number of embryos [6, 22].

The transcriptome analysis was performed at three specific time points of embryo development: day 4.5, 8 and 12. According to the available literature, the germ cells of most species undergo two complex developmental phases [23]. The first phase occurs during early embryogenesis. PGCs are formed in this phrase, and they then actively migrate to the gonads. In the second phase, the germ cells initiate one of the two distinct programs of cell division: meiosis and differentiation—oogenesis or spermatogenesis to form gametes [24]. However, very little is known about the molecular mechanisms that govern these programs. Therefore, we used the transcriptome analysis to detect dynamic changes of gene expression in gPGCs at three developmental stages. According to Swartz and Domm [25], an increase in the number of PGCs is observed during the embryonic period from the start of migration to 5 days of incubation. During this period, the number of PGCs ranged from 43 to 2211. A period of intense proliferation is observed between the fourth and fifth day of embryo development. Therefore, the first data point for the transcriptome analysis of PGCs was set at day 4.5 of embryo development. Méndez et al. [26] indicated the variation in the time of growth in chick gonad depends on the sex of the embryo. At 8 days of incubation, the left ovary initiates a period of exponential growth, as shown by the increase in the total number of somatic cells. A similar pattern of increment was observed in the number of germ cells of the chick embryo ovary. This was the reason for selecting day 8 as the second data point for the transcriptome analysis of PGCs. The last data point, i.e. day 12, was selected because of somatic conversion [27]. This process involves rearrangement of immunoglobulin genes. In birds, it occurs only once during the entire lifetime in the course of embryonic development.

The present study showed the highest number of upregulated DEGs on day 12 of embryo development. The expression of these genes is mainly associated with biological pathways such as multicellular organismal processes, developmental process and anatomical structure development. On day 8 of embryo development, a significant decrease in gene expression was observed. We assume that there is a negative regulation in the 8th day in relation to day 4.5, because there is no more increased cell migration and proliferation that may take place on day 4.5. Significant upregulation at day 12 is likely to be associated with a number of immune processes. Also, environmental effects of CpG island methylation, which may lead to gene expression silencing, cannot be clearly excluded [28]. The common denominator for the observed relationships among the upregulated genes on day 8 is the *SPARC* gene. This protein regulates cell growth by interacting with the extracellular matrix and cytokines. The relationships among genes with negative expression in PGCs on day 8 of embryo development relative to day 4 are derived from the *SERPINB10* and *INS* genes. *SERPINB10* plays a role in regulating protease activity during apoptosis. *INS* plays an important role in carbohydrate and lipid metabolism by increasing cell permeability to monosaccharides, amino acids and fatty acids.

These results shed new light on the transcriptome analysis of PGCs, treating them as a tool for the global view of embryonic development. Changes in gene expression depend on the time point of PGCs isolation during embryonic development. This study shows that on all the analysed days, i.e. 4.5, 8 and 12, PGCs are present in the gonads and show significant transcriptional activity.

### Limitations

The present study aimed to show how quantitative gene expression in PGCs changes during chicken embryonic development. However, we did not determine the sex of PGC embryo donors. Further studies are needed to clarify the effect of the sex of the embryo on the transcriptome of chicken PGCs.

## Supplementary information

**Additional file 1: Figure S1.** Migration of chicken PGCs in early embryogenesis (according to Kuwana T., 2018, unpublished)**. Figure S2.** *NANOG* gene amplification curve based on RNA isolated from gonadal PGCs collected from 3 stages of embryonic development: 4.5, 8 and 12.**. Figure S3.** *DAZL* gene amplification curve based on RNA isolated from gonadal PGCs collected from 3 stages of embryonic development: 4.5, 8 and 12. **Figure S4.** The number of differentially expressed genes showing up regulation (cut off >2.0) of expression in primordial germs cells detected between embryo development days:12 vs 4.5 (blue), 8 vs 4.5 (red) and 8 vs 12 (green). **Figure S5.** The number of differentially expressed genes showing down regulation (cut off >2.0) of expression in primordial germs cells detected between embryo development days:12 vs 4.5 (blue), 8 vs 4.5 (red) and 8 vs 12 (green)**. Figure S6.** Analysis of the relationship between proteins encoded by genes whose expression was downregulated in PGCs on day 8 of embryo development. Lines of interactions according to STING: light blue – from curated databases; pink – experimentally determined; dark green – gene neighborhood; red – gene fusions; dark blue – gene co-occurrence; light green – textmining; black – co-expression; violett – protein homology. **Table S1.** List of selected genes for microarray validation with designed primer sequences for RT-qPCR reaction. **Table S2.** Microarray validation. Results of RT-qPCR analysis for panel of the most up or down expressed genes selected from microarrays results in 3 stages of embryo development. *statistically significant (*P* < 0.05).

## Data Availability

The datasets used and/or analyzed during the current study are available from the corresponding author on reasonable request.

## Abbreviations

PGCs: Primordial germ cells
cPGCs: Circulating primordial germ cells
gPGCs: Gonadal primordial germ cells
DEGs: Differentially expressed genes
Ct: Threshold cycle
RT-qPCR: Reverse transcription-quantitative polymerase chain reaction
