# Supplementary material for: Dynamics of the transcriptome during chicken embryo development based on primordial germ cells
Source: BMC Res Notes. 2020 Sep 18;13:441. doi: 10.1186/s13104-020-05286-w (PMC7501632; doi:10.1186/s13104-020-05286-w)
Supplement: Supplementary file 1 — Additional file 1: Figure S1. Migration of chicken PGCs in early embryogenesis (according to Kuwana T., 2018, unpublished). Figure S2. NANOG gene amplification curve based on RNA isolated from gonadal PGCs collected from 3 stages of embryonic development: 4.5, 8 and 12.. Figure S3. DAZL gene amplification curve based on RNA isolated from gonadal PGCs collected from 3 stages of embryonic development: 4.5, 8 and 12. Figure S4. The number of differentially expressed genes showing up regulation (cut off >2.0) of expression in primordial germs cells detected between embryo development days:12 vs 4.5 (blue), 8 vs 4.5 (red) and 8 vs 12 (green). Figure S5. The number of differentially expressed genes showing down regulation (cut off >2.0) of expression in primordial germs cells detected between embryo development days:12 vs 4.5 (blue), 8 vs 4.5 (red) and 8 vs 12 (green). Figure S6. Analysis of the relationship between proteins encoded by genes whose expression was downregulated in PGCs on day 8 of embryo development. Lines of interactions according to STING: light blue – from curated databases; pink – experimentally determined; dark green – gene neighborhood; red – gene fusions; dark blue – gene co-occurrence; light green – textmining; black – co-expression; violett – protein homology. Table S1. List of selected genes for microarray validation with designed primer sequences for RT-qPCR reaction. Table S2. Microarray validation. Results of RT-qPCR analysis for panel of the most up or down expressed genes selected from microarrays results in 3 stages of embryo development. *statistically significant (P < 0.05). [file 13104_2020_5286_MOESM1_ESM.docx]

Additional file 1

4 h 18 h 23 h 33 h 48 h 72 h

← Circulation phase → ← gonadal phase →

Figure S1. Migration of chicken PGCs in early embryogenesis (according to Kuwana T., 2018, unpublished)

*
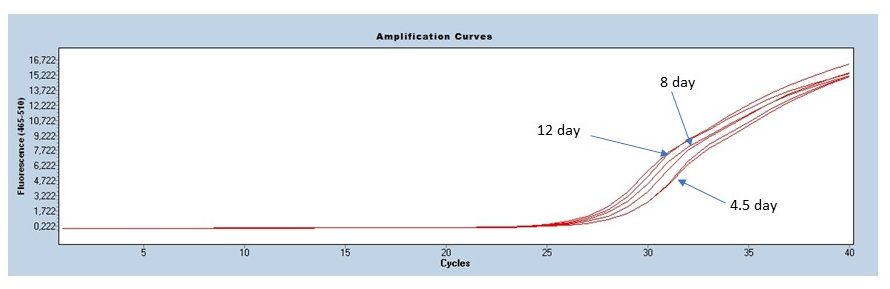
*

Figure S2. *NANOG* gene amplification curve based on RNA isolated from gonadal PGCs collected from 3 stages of embryonic development: 4.5, 8 and 12.

*
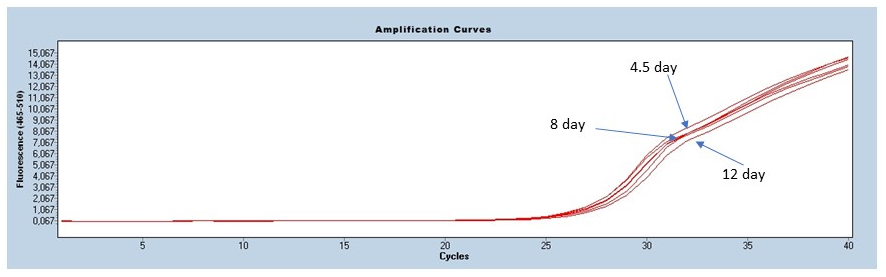
* Figure S3. *DAZL* gene amplification curve based on RNA isolated from gonadal PGCs collected from 3 stages of embryonic development: 4.5, 8 and 12.


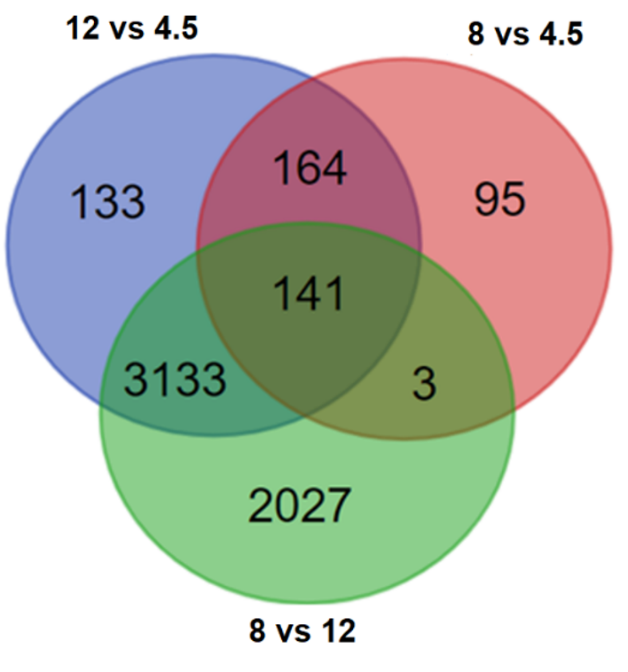


**Figure S4.** The number of differentially expressed genes showing up regulation (cut off >2.0) of expression in primordial germs cells detected between embryo development days:12 vs 4.5 (blue), 8 vs 4.5 (red) and 8 vs 12 (green).


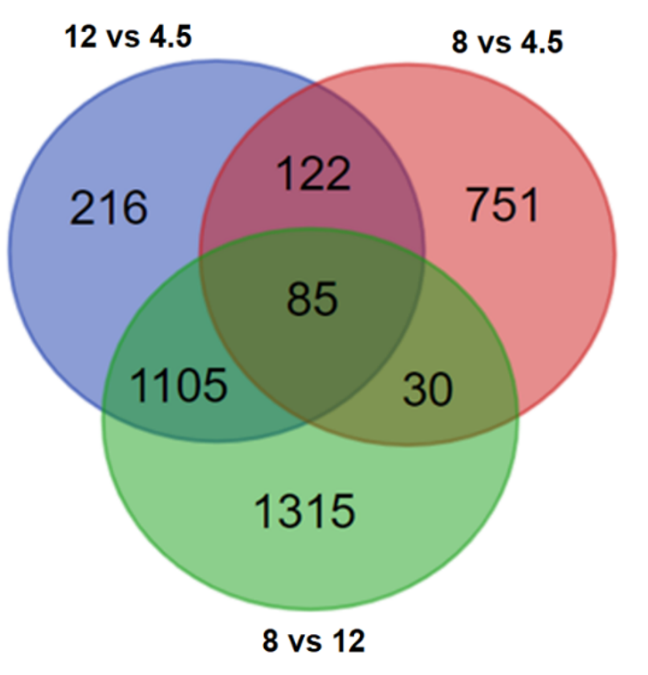


**Figure S5.** The number of differentially expressed genes showing down regulation (cut off >2.0) of expression in primordial germs cells detected between embryo development days:12 vs 4.5 (blue), 8 vs 4.5 (red) and 8 vs 12 (green).


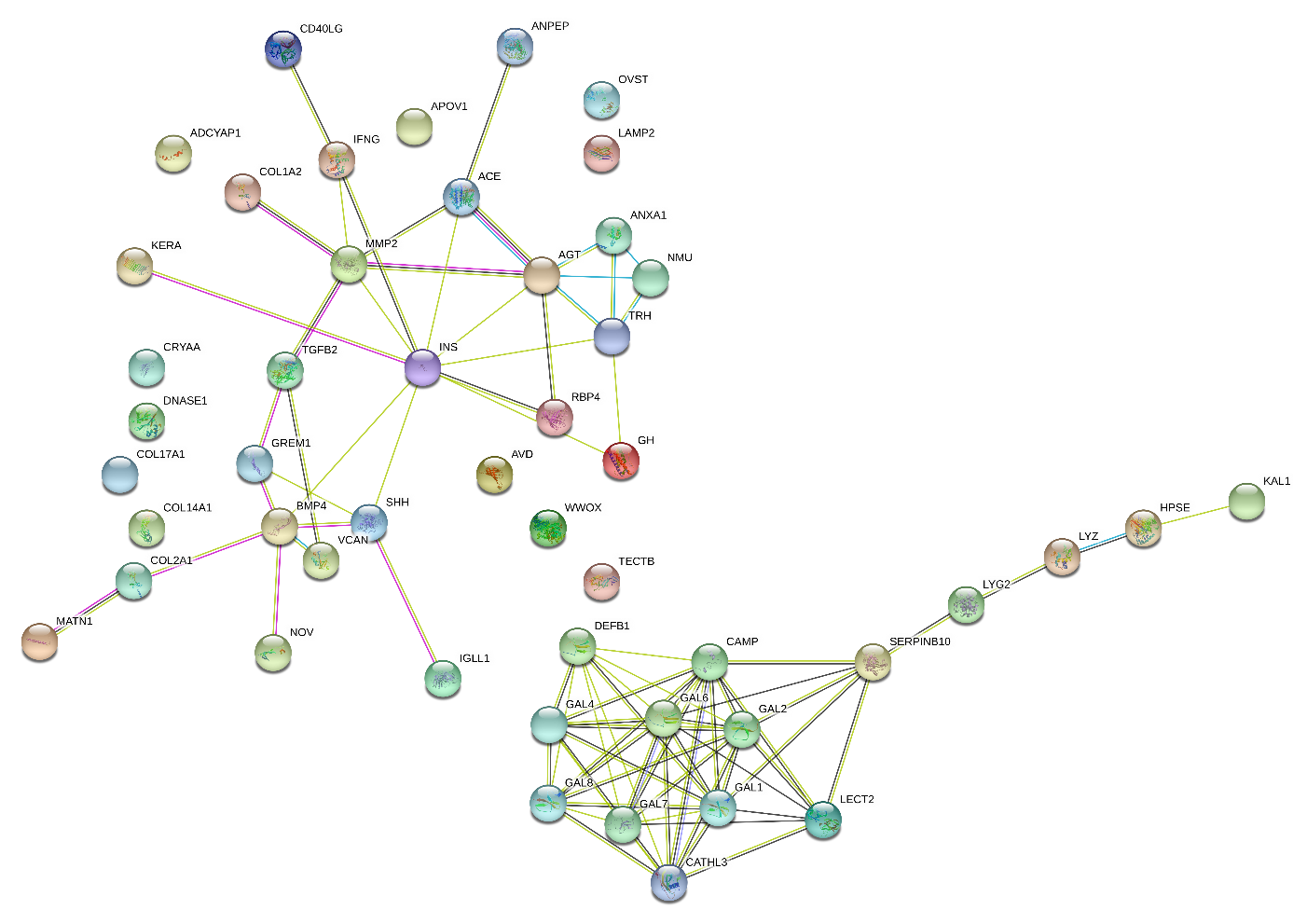


**Figure S6.** Analysis of the relationship between proteins encoded by genes whose expression was downregulated in PGCs on day 8 of embryo development. Lines of interactions according to STING: light blue – from curated databases; pink – experimentally determined; dark green – gene neighborhood; red – gene fusions; dark blue – gene co-occurrence; light green – textmining; black – co-expression; violett – protein homology.

**Table S1**. List of selected genes for microarray validation with designed primer sequences for RT-qPCR reaction

| Gene symbol | NCBI ID | Sequence (Forward/Reverse) | Product length |
| --- | --- | --- | --- |
| CYP11A1 | 414838 | F: CTGCAGAGGTACACCACACA  R: ATATGATTCCGTGACCCACCC | 456 |
| CCL4 | 395468 | F: AGCGTAGGAACTCCACTCTCT  R: ATCGGTACCTACATGATGGCAG | 302 |
| ANXA1 | 404271 | F: CAGGCTGGACGTCAGGAAAT  R: CCAGGGACCACCTAGATTCC | 347 |
| AvBD1 | 395841 | F: ACAAGCCACCTCCTGGGATA  R: TGTATCTTGTGTGGCCTTGGT | 173 |
| SOHO1 | 396346 | F: CTTCACGTCGAAGGTGGACT  R: CTGTGTTTTGCCATTGCAGGC | 252 |
| MYH7 | 395350 | F: GACGCTCTGTCCTTTGGTGA  R: GGCACTGAAGGTTCATGGGA | 217 |
| NEFL | 419528 | F: AGGTGAAGAGACCCGACTCA  R: TATGAGCAGGGTGAAGCATGG | 364 |
| MIEN1 | 100858225 | F: CTCCCACGGGAACACAAAGA  R: GCTTTTGGGTTCACTCCCAC | 184 |

**Table S2.** Microarray validation

Results of RT-qPCR analysis for panel of the most up or down expressed genes selected from microarrays results in 3 stages of embryo development. *statistically significant (*P* < 0.05).

| Ensembl ID | Gene name | LOG Fold change (microarray) | LOG Fold induction (qPCR) | Description | Function |
| --- | --- | --- | --- | --- | --- |
| UP 8 vs 4 | | | | |  |
| ENSGALT00000055423 | CYP11A1 | 4.11 | -1.49 | cytochrome P450 family 11 subfamily A member 1 | catalyzes the side-chain hydroxylation and cleavage of cholesterol to pregnenolone, the precursor of most steroid hormone |
| ENSGALT00000053861 | CCL4 | 3.7 | 0.31 | C-C motif chemokine ligand 4 | monokine with inflammatory and chemokinetic properties |
| DOWN 8 vs 4 | | | | |  |
| ENSGALT00000032821 | ANXA1 | -4.12 | -1.01 | annexin A1 | important roles in the innate immune response as effector of glucocorticoid-mediated responses and regulator of the inflammatory process. Has anti-inflammatory activity |
| ENSGALT00000036713 | AvBD1 | -3.89 | -0.03 | avian beta-defensin 1 | defense response |
| UP 12 vs 4 | | | | |  |
| ENSGALT00000025152 | SOHO1 | 4.8 | 0.91 | sensory organ homeobox protein SOHo | regulation of transcription, DNA-templated |
| DOWN 12 vs 4 | | | | |  |
| ENSGALT00000054723 | MYH7 | -3.9 | -1.55* (*P*<0.05) | myosin, heavy chain 7, cardiac muscle, beta | actin filament binding, ATP binding, motor activity |
| ENSGALT00000087263 | NEFL | -4.19 | 0.25 | neurofilament, light polypeptide | maintain the neuronal caliber, play a role in intracellular transport to axons and dendrites |
| ENSGALT00000045413 | MIEN1 | -2.52 | -1.86* (*P*<0.05) | migration and invasion enhancer 1 | increases cell migration by inducing filopodia formation at the leading edge of migrating cells, plays a role in regulation of apoptosis |
